# Altered Nucleosome Positioning at the Transcription Start Site and Deficient Transcriptional Initiation in Friedreich Ataxia*

**DOI:** 10.1074/jbc.M114.566414

**Published:** 2014-04-15

**Authors:** Yogesh K. Chutake, Whitney N. Costello, Christina Lam, Sanjay I. Bidichandani

**Affiliations:** From the Departments of ‡ Pediatrics and; § Biochemistry & Molecular Biology, University of Oklahoma Health Sciences Center, Oklahoma City, Oklahoma 73104

**Keywords:** Gene Transcription, Heterochromatin, Neurological Diseases, Nucleotide Repeat Disease, Transcription Promoter, Friedreich Ataxia, Expanded Triplet Repeat, Nucleosome Positioning, Transcription Start Site

## Abstract

Most individuals with Friedreich ataxia (FRDA) are homozygous for an expanded GAA triplet repeat (GAA-TR) mutation in intron 1 of the *FXN* gene, which results in deficiency of *FXN* transcript. Consistent with the expanded GAA-TR sequence as a cause of variegated gene silencing, evidence for heterochromatin has been detected in intron 1 in the immediate vicinity of the expanded GAA-TR mutation in FRDA. Transcriptional deficiency in FRDA is thought to result from deficient elongation through the expanded GAA-TR sequence because of repeat-proximal heterochromatin and abnormal DNA structures adopted by the expanded repeat. There is also evidence for deficient transcriptional initiation in FRDA, but its relationship to the expanded GAA-TR mutation remains unclear. We show that repressive chromatin extends from the expanded GAA-TR in intron 1 to the upstream regions of the *FXN* gene, involving the *FXN* transcriptional start site. Using a chromatin accessibility assay and a high-resolution nucleosome occupancy assay, we found that the major *FXN* transcriptional start site, which is normally in a nucleosome-depleted region, is rendered inaccessible by altered nucleosome positioning in FRDA. Consistent with the altered epigenetic landscape the *FXN* gene promoter, a typical CpG island promoter, was found to be in a transcriptionally non-permissive state in FRDA. Both metabolic labeling of nascent transcripts and an unbiased whole transcriptome analysis revealed a severe deficiency of transcriptional initiation in FRDA. Deficient transcriptional initiation, and not elongation, is the major cause of *FXN* transcriptional deficiency in FRDA, and it is related to the spread of repressive chromatin from the expanded GAA-TR mutation.

## Teaser

**Background:** Friedreich ataxia (FRDA) is caused by an expanded GAA triplet repeat (GAA-TR) mutation that results in *FXN* transcriptional deficiency.

**Results:** Repressive chromatin spreads from the expanded GAA-TR mutation, switching off the *FXN* gene promoter.

**Conclusion:** Reduced transcriptional initiation is the major cause of *FXN* transcriptional deficiency in FRDA.

**Significance:** Reactivation of *FXN* transcriptional initiation is a potential therapeutic strategy in FRDA.

## Introduction

Friedreich ataxia (FRDA)^2^ is the most common inherited ataxia, and it is characterized clinically by progressive sensory ataxia, cardiomyopathy, and a predisposition to diabetes (1). The disease is variably progressive, and there are no effective therapies currently available to slow the inevitable phenotypic deterioration. FRDA is inherited as an autosomal recessive condition, and the vast majority of patients are homozygous for an abnormally expanded GAA triplet repeat (GAA-TR) sequence in intron 1 of the *FXN* gene (2). Whereas normal alleles contain <30 triplets, disease-causing expanded alleles typically contain 100–1700 triplets. Cells and tissues from patients who are homozygous for the expanded GAA-TR sequence show a severe deficiency of *FXN* transcript (3). This ultimately leads to a deficiency of frataxin, a mitochondrial protein that plays an important role in Fe-S cluster biogenesis and in mitochondrial iron metabolism (4). The precise delineation of the mechanism(s) by which the expanded GAA-TR results in transcriptional deficiency will be crucial for the development of rationally designed therapies for FRDA.

Saveliev *et al.* (5) made the seminal discovery that expanded triplet repeats, including the expanded GAA-TR sequence, can result in epigenetic silencing of a closely linked transgene via a mechanism resembling position effect variegation. They showed that the expanded GAA-TR served as a source of HP-1-mediated heterochromatin, which was sufficient to silence a nearby reporter gene. Several groups have since identified molecular signatures of heterochromatin in the vicinity of the expanded GAA-TR in intron 1 of the *FXN* gene in cells and tissues from FRDA patients (6–13), including histone deacetylation (H3K9Ac, H3K14Ac, and H4K5Ac), histone trimethylation (H3K9me3 and H3K27me3), and CpG DNA methylation. Although these changes were seen both upstream and downstream of the expanded GAA-TR, evidence of heterochromatin was most pronounced immediately upstream of the GAA-TR but still within intron 1. Importantly, Herman *et al.* (6) showed that derivatives of a 2-aminobenzamide histone deacetylase inhibitor were able, albeit partially, to reverse both the epigenetic defect and the transcriptional silencing in lymphoid cells from FRDA patients. Indeed, a lead compound based on this histone deacetylase inhibitor is currently in clinical development as a rational therapeutic agent for Friedreich ataxia.

The expanded GAA-TR sequence is also known to adopt abnormal structures such as the triplex-based “sticky” DNA (14) and R-loops (15), both of which have the ability to interfere with transcriptional elongation. Indeed, destabilization of triplex-based structures via polyamides resulted in partial reversal of the transcriptional deficiency in lymphoblastoid cells from FRDA patients (16). The expanded GAA-TR in intron 1 does not result in abnormal splicing (3), resulting instead in lower quantities of a normally spliced *FXN* transcript in FRDA with a normal half-life (10). Taken together, these observations support the model that *FXN* transcriptional deficiency in FRDA is due to deficient transcriptional elongation, mediated via heterochromatin in intron 1 in the immediate vicinity of the expanded GAA-TR and possibly also one or more abnormal structures adopted by the expanded GAA-TR.

Interestingly, there is also evidence of transcriptional deficiency and of markers of repressive chromatin located further upstream from the changes noted in the immediate vicinity of the expanded GAA-TR in intron 1. For instance, in fibroblast cell lines from FRDA patients, markers of repressive chromatin were detected at the *FXN* transcriptional start site (*FXN*-TSS) (17). Furthermore, reduced RNAPII occupancy (12) and deficiency of *FXN* transcript (11) were noted in exon 1, *i.e.* considerably upstream of the expanded GAA-TR in intron 1. Indeed, Kumari *et al.* (11) also demonstrated reduced levels of serine 5-phosphorylated RNAPII, *i.e.* the initiating form of RNAPII, near the *FXN* promoter in patient-derived cell lines. These findings clearly support the existence of an additional transcriptional defect in FRDA: one that involves transcriptional initiation (or immediate post-initiation) of the *FXN* gene. However, the relative magnitude and the mechanism of this initiation defect in FRDA remain unclear, and its relationship to the expanded GAA-TR in intron 1 also remains unknown.

Heterochromatin, once formed, tends to spread to contiguous DNA sequence with some facility. Repressive chromatin associated with variegated silencing caused by expanded triplet repeats was found to spread over at least a few thousand bases (5). Given that the distance between the expanded GAA-TR in intron 1 and the *FXN*-TSS (and *FXN* promoter) is <2 kb, we decided to characterize the distance over which repressive chromatin spreads upstream of the expanded GAA-TR in FRDA. We further sought to determine whether the *FXN* gene promoter had been functionally and structurally altered so as to result in deficiency of transcriptional initiation.

We found that in FRDA repressive chromatin extends from the expanded GAA-TR in intron 1 toward the promoter region of the *FXN* gene and encompasses the *FXN*-TSS. The *FXN*-TSS is thus rendered inaccessible via altered nucleosome positioning that obliterates the nucleosome-depleted region (NDR) within which *FXN* transcriptional initiation is known to occur. The *FXN* promoter in FRDA is thus switched to a transcriptionally non-permissive state, which results in a severe deficiency of transcriptional initiation. Indeed, deficient transcriptional initiation, and not elongation, seems to be the major cause of *FXN* transcriptional deficiency in FRDA. These data have important implications for the pathogenesis and treatment of FRDA and perhaps also for other diseases caused by expanded triplet repeats.

## EXPERIMENTAL PROCEDURES

### 

#### 

##### Cell Lines

Most experiments involved the comparison of three lymphoblast cell lines from individuals with FRDA *versus* three cell lines from healthy subjects. Lymphoblast cell lines from three healthy subjects, GM15851, GM22671, GM22647, and two individuals with FRDA, GM15850 (650 and 1030 repeats) and GM16209 (800 and 800 repeats), were obtained from Coriell Cell Repositories. An additional FRDA lymphoblast cell line, OK22 (400 and 860 repeats), was established in the laboratory. However, in NOMe-Seq and RNA-Seq assays, we compared two FRDA *versus* two non-FRDA lymphoblast cell lines; in these assays, FRDA-1 and FRDA-2 refer to GM15850 and OK22, and CNTR-1 and CNTR-2 refer to GM22647 and GM22671, respectively.

##### Chromatin Immunoprecipitation (ChIP)

Chromatin for conventional ChIP assays was prepared according to ChIP-IT® Express Sonication Shearing Kit (Active Motif) with some modifications. Briefly, 6 million cells were cross-linked with 1% freshly prepared formaldehyde. Cross-linked nuclei were sheared with the EpiShear^TM^ sonicator (Active Motif) to yield a chromatin size range of 200 to 800 bp. Immunoprecipitation was performed using anti-RNAPII (Millipore, 17-620), anti-H2A.Z (Millipore, 17-10048), and anti-histone H3 (Millipore, 17-10254). Immunoprecipitated chromatin-bead complexes were washed and transferred to new tubes for elution to minimize contamination with non-specifically bound chromatin to the tube surface. Immunoprecipitated chromatin was eluted from the beads, reverse cross-linked, treated with proteinase K, and extracted using phenol-chloroform. The immunoprecipitated DNA and the input DNA were analyzed by real-time PCR using the ΔΔ*C_t_* method using an Eppendorf RealPlex-4 Mastercycler with Power SYBR Green PCR Mastermix (Applied Biosystems).

Chromatin for mononucleosomal ChIP was prepared using the ChIP-IT® Express Enzymatic Shearing Kit (Active Motif). Mononucleosomal preparations (size ∼ 150 bp) were obtained by optimizing the digestion reaction with enzymatic shearing mixture. Immunoprecipitation was performed using anti-H3K27me3 (Millipore, 17-622) and anti-H4K5Ac (Millipore, 07-327).

Each value of immunoprecipitated DNA was normalized to the corresponding input DNA. For conventional ChIP, relative recovery was calculated over histone H3 enrichment corresponding to the region being analyzed. All quantitative PCRs were performed in a two-step cycle with an annealing/extension temperature of 62 °C (see Table 1 for primer sequences).

**TABLE 1:** **Primer sequences used in this study** *, primers were also used for strand-specific reverse transcription.

Primer	Forward (5′ to 3′)	Reverse (5′ to 3′)
**Primers used for chromatin immunoprecipitation**		
P4 (−2632 to −2513)	CCAAGGAAGTGGAGAAAGACAAG	TCCAGCTCCCTGCAAAGTTG
P3 (−1719 to −1602)	CCCTGGACCACCTCTTTGATAG	GAGCTAGGGATGGGGGAATC
P2 (−1261 to −1134)	CGGCTCACATTTGACATCCTC	GTGCCTTGCCTATTGTTGATAGAT
P1 (−358 to −232)	CCCCACATACCCAACTGCTG	GCCCGCCGCTTCTAAAATTC
U1 (−204 to −77)	GGGGTCCTGGTTGCACTCC	GCACTCTTCTGTGGGGGAGC
E1 (−46 to +59)	AGCACCCAGCGCTGGAGG	TGGGCTGGGCTGGGTGACGCCAGG
I1 (+277 to +396)	ACTAGCTCACCCCGCTCCT	AAATGTCCCCTTTTCCTTCG
I2 (+666 to +778)	TGAACGAAATGCTTTCCTGG	GGGCAGAATCTGGAATAAAGG
I3 (+1006 to +1121) (Ref. 6)	GAAACCCAAAGAATGGCTGTG	TTCCCTCCTCGTGAAACACC

**Primers used for RT-PCR**		
Ex1 (Ref. 11)	AGCAGCATGTGGACTCTC	TGGGCTGGGCTGGGTGACGCCAGG*
Ex1-In1	CCGACATCGATGCGACCTGC	GTTCCCGGCGCGGATACTTACT
Ex2-Ex4	CCGCGCAAGTTCGAACCAA	TTCCTAGATCTCCACCCAGT
RPS29	GCACTGCTGAGAGCAAGATG	ATAGGCAGTGCCAAGGAAGA

**Primers used for NOMe-Seq**		
Amplicon 1	CCTATCCAAAAAAAACTTTACAAAAC	GGTTAGATTTTAGGAAGAATTTTTT
Amplicon 2	AATCTCCCTTAAATCAAAAATCC	GATTTGGGTGAGGGTTTGGGTTTGG

**Primers used for chromatin accessibility assay**		
*FXN*-TSS2	TCCTGAGGTCTAACCTCTAGCTGC	TGGGCTGGGCTGGGTGACGCCAGG

##### Quantitative RT-PCR

Total RNA was isolated using the RNeasy mini kit (Qiagen). Reverse transcription was performed either with a mixture of random hexamers and oligo(dT) primers or strand-specific primers using the QuantiTect® Reverse Transcription kit (Qiagen). mRNA levels were quantified by real-time PCR relative to RPS29 using the ΔΔ*C_t_* method using an Eppendorf RealPlex-4 Mastercycler with SsoAdvanced^TM^ SYBR® Green supermix (Bio-Rad). Quantitative PCRs were performed in a two-step cycle with an annealing/extension temperature of 70 °C (see Table 1 for primer sequences).

##### Chromatin Accessibility Assay

The EpiQ^TM^ chromatin analysis kit (Bio-Rad) was used with minor modifications. For each cell line, a set of two 200,000 cell aliquots were analyzed; one aliquot was treated with nuclease (37 °C for 30 min), and the other was used as an undigested control. 5 ng of DNA isolated from each sample was used for quantitative PCR, done in triplicate to determine % accessibility, which was calculated using the data analysis tool available from Bio-Rad. The β hemoglobin (HBB) gene was used as the inaccessible reference for measuring relative chromatin accessibility at various gene loci. The GAPDH gene was used as a constitutively accessible control, and the RHO gene was used as the inaccessible control. PCR primer sequences for HBB, GAPDH, and RHO are as provided in the EpiQ^TM^ chromatin analysis kit. The region of the *FXN* gene analyzed is shown in Fig. 2*A*, and the PCR primer sequences are listed in Table 1.

##### Nucleosome Occupancy and Methylome Sequencing (NOMe-Seq)

NOMe-Seq was performed according to the manufacturer's instructions (Active Motif) with a few modifications. Briefly, 750,000 cells were cross-linked as described above. Chromatin was sheared by sonication on ice with 25% amplitude for 3 min of 30 s “on” and 30 s “off” cycle. The GpC methyltransferase reaction was performed on sheared chromatin from ∼250,000 cells. 1–2 μg of the GpC methyltransferase-treated DNA was subjected to bisulfite conversion. 20–50 ng of each bisulfite-treated DNA was used for PCR amplification (see Table 1 for primer sequences of the two overlapping amplicons analyzed) using AmpliTaq® Gold DNA polymerase (Applied Biosystems). PCR products were TA-cloned using the TOPO TA Cloning® Kit (Invitrogen) and transformed into competent *Escherichia coli* (DH5α), and individual clones were sequenced. Cytosines at GpC sites that are accessible to GpC methyltransferase (*i.e.* not within a nucleosome) are methylated and therefore not converted by bisulfite treatment (*i.e.* read as cytosine) and are denoted as a *white box* in Fig. 3. Conversely, inaccessible cytosines, which remain unmethylated after GpC methyltransferase treatment, are converted via bisulfite treatment to uracil (*i.e.* read as thymine) and are denoted as a *black box* in Fig. 3. The discriminatory capacity of the assay relies on the efficiency of bisulfite conversion; therefore, only clones that demonstrated complete conversion efficiency at all non-GpC (and non-CpG) sites were used for NOMe-Seq analysis (>98% conversion of all non-GpC sites was attained for the clones shown in Fig. 3).

##### RNA-Seq/Transcriptome Analysis

Total RNA was extracted from two non-FRDA (GM22647 and GM22671) and two FRDA (GM15850 and OK22) lymphoblast cell lines. Strand-specific libraries were generated from poly-A-selected RNA. Multiplexed libraries were paired end-sequenced (100-bp reads) on the Illumina HiSeq 2500 system for a total of 2 × 20–30 million reads per sample. The sequence quality as calculated by % of ≥Q30 bases (PF) was 92.45, 92.66, 92.09, and 92.49 for GM22647 (CNTR-1), GM22671 (CNTR-2), GM15850 (FRDA-1), and OK22 (FRDA-2), respectively.

##### Metabolic Labeling of Nascent RNA in Live Cells

Metabolic labeling of nascent RNA was performed using the Click-iT® Nascent RNA Capture Kit (Invitrogen). Briefly, lymphoblastoid cells at a density of 1 million/ml were incubated with 0.2 mm 5-ethynyl uridine. 2 million cells were removed after 0.5, 1, 2, and 4 h of 5-ethynyl uridine treatment. RNA was extracted using the TriPure Isolation Reagent (Roche Applied Science). 1 μg of 5-ethynyl uridine RNA was used for biotinylation by Click reaction. After purification, 250 ng of biotinylated RNA was used for pulldown with streptavidin beads. Biotinylated RNA bound to beads were serially washed, and the bound RNA was reverse-transcribed using the SuperScript® VILO^TM^ cDNA synthesis kit (Invitrogen) following the manufacturer's instructions. The mRNA levels were quantified by real-time PCR relative to RPS29 from total biotinylated RNA using the ΔΔ*C_t_* method on the Eppendorf RealPlex-4 Mastercycler with SsoAdvanced SYBR Green Supermix (Bio-Rad). All quantitative PCRs were performed in a two-step cycle with an annealing-extension temperature of 70 °C (see Table 1 for primer sequences).

##### Statistical Methods

Statistical significance was calculated using paired, two-tailed Student's *t* test. *p* < 0.05 was considered significant.

## RESULTS

### 

#### 

##### Repressive Chromatin Extends from the Expanded GAA Repeat in Intron 1 toward the FXN Gene Promoter and Encompasses the FXN-TSS in FRDA

**FIGURE 1.**
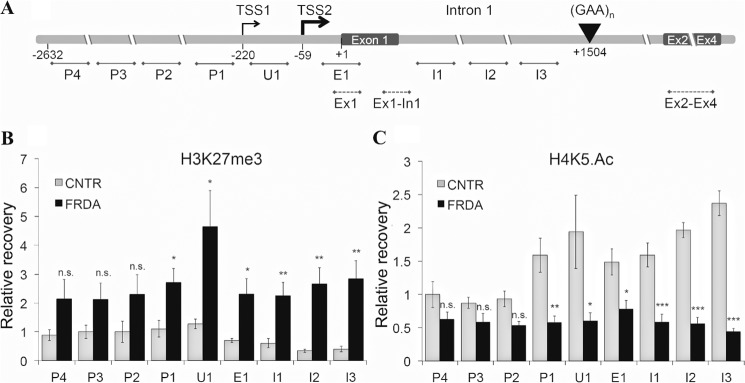
**Repressive chromatin extends from the expanded GAA-TR in intron 1 to the *FXN*-TSS in FRDA.** *A*, the relevant portion of the *FXN* gene is shown, with the GAA-TR sequence in intron 1 and the positions of the major and minor *FXN* transcriptional start sites (TSS2 at −59 and TSS1 at −220, respectively). Regions depicted with *solid lines* were evaluated by ChIP. Of these, three amplicons spanned the relevant portions of intron 1 (I1–3), one sampled the 5′-UTR/exon 1 (E1; immediately downstream of *FXN*-TSS2), one sampled the 5′-UTR (U1; in between the two reported *FXN*-TSS) and four amplicons covered the putative *FXN* promoter up to 2.6 kb upstream of the *FXN*-TSS (P1–4). Regions depicted with *dotted lines* were analyzed by quantitative RT-PCR. All location numbers are relative to the *A* (+1) in the initiation codon. *B* and *C*, mononucleosomal ChIP showing enrichment for H3K27me3 (*B*), and hypoacetylation of H4K5 (*C*) extending from intron 1 (I3) up to and including the *FXN*-TSS. These data represent the cumulative results from two complete experiments using three FRDA and three non-FRDA control (*CNTR*) lymphoblast cell lines, each assayed in triplicate. The *x* axis labels refer to similarly labeled regions depicted in *A. Error bars* represent ±S.E. *, *p* < 0.5; **, *p* < 0.01; ***, *p* < 0.001; *n.s.*, not significant.

The 5′ region of the *FXN* gene spanning the putative promoter, the *FXN*-TSS, 5′-UTR, exon 1, and the sequence upstream of the expanded GAA-TR in intron 1 (Fig. 1*A*) were analyzed by chromatin immunoprecipitation for markers of repressive chromatin. Lymphoblast cell lines from three FRDA patients, each homozygous for the expanded GAA-TR sequence (range, 400–1030 triplets), and three non-FRDA controls, were analyzed for enrichment of H3K27me3 and hypoacetylation of H4K5, *i.e.* known markers of repressive chromatin. FRDA patients showed both enrichment of H3K27me3 and hypoacetylation of H4K5 for regions I3 through P1 (Fig. 1, *B* and *C*), indicating the presence of repressive chromatin spanning the region of the *FXN* gene from the expanded GAA-TR in intron 1 to the *FXN*-TSS. The extended *FXN* promoter region, spanning up to 2.6 kb upstream of the *FXN*-TSS, seemed to be spared in FRDA. These data support the model that in FRDA repressive chromatin spreads upstream from the expanded GAA-TR in intron 1 to encompass the *FXN*-TSS.

##### Reduced Accessibility of the FXN-TSS in FRDA via Altered Nucleosome Positioning and Obliteration of the Nucleosome-depleted Region of the FXN Gene Promoter

**FIGURE 2.**
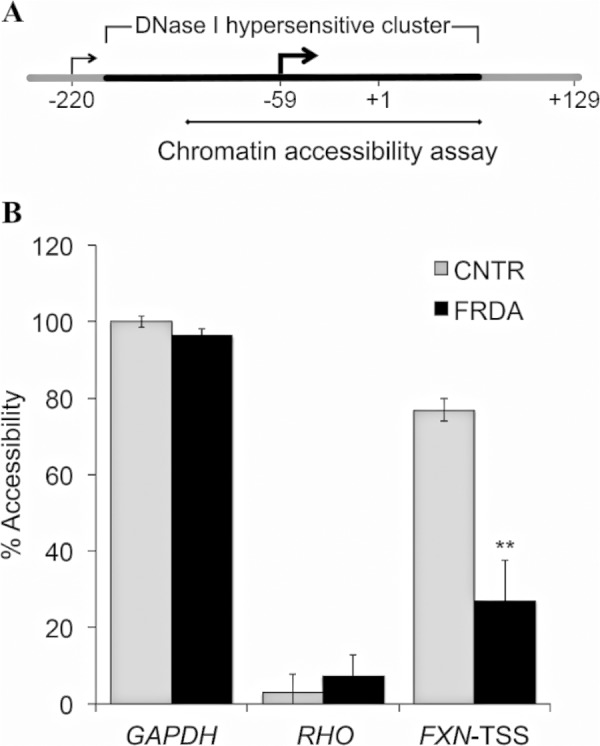
**Reduced chromatin accessibility at the major *FXN*-TSS in FRDA.** *A*, the major *FXN*-TSS (*FXN*-TSS2 at −59), which maps within an NDR (DNase I-hypersensitive cluster in ENCODE), is depicted with an *arrow* in *boldface type*. The region analyzed for chromatin accessibility, which includes *FXN*-TSS2, is indicated by a *horizontal line. B*, relative chromatin accessibility (%) is significantly reduced in FRDA *versus* non-FRDA (CNTR) cells. Two control regions are shown that do not show any difference in chromatin accessibility in FRDA *versus* non-FRDA cells; a constitutively accessible region of the GAPDH gene, and a region of the RHO gene that is known to be inaccessible. These data represent the cumulative results from two complete experiments using three FRDA and three non-FRDA (CNTR) lymphoblast cell lines, with each assayed in triplicate. *Error bars* represent ± S.E. **, *p* < 0.01.

To test whether the repressive chromatin in FRDA results in reduced accessibility of the *FXN*-TSS, a quantitative chromatin accessibility assay was performed based on measurement of nuclease sensitivity in live cells. As is typical for most human CpG island promoters (18), the major TSS of the *FXN* gene, *FXN*-TSS2 (position −59; Fig. 2*A*) is known to map within an NDR (DNase I-hypersensitive cluster in ENCODE; Fig. 2*A*). Indeed, *FXN*-TSS2 is likely the only active *FXN*-TSS in lymphoblasts (11); also see transcriptome analysis below). Chromatin accessibility at *FXN*-TSS2 was measured in three FRDA and three non-FRDA lymphoblast cell lines. Although it was highly accessible in non-FRDA cells, *FXN*-TSS2 showed significantly reduced chromatin accessibility in FRDA patients (Fig. 2*B*). In contrast, a constitutively accessible site in the *GAPDH* gene was found to be highly accessible, and a region of the gene encoding rhodopsin that is inaccessible in all cells except photoreceptors was found to be highly inaccessible, with no noticeable difference in accessibility between FRDA and non-FRDA cell lines (Fig. 2*B*). This indicates that in FRDA, repressive chromatin results in reduced chromatin accessibility at the *FXN*-TSS.

**FIGURE 3.**
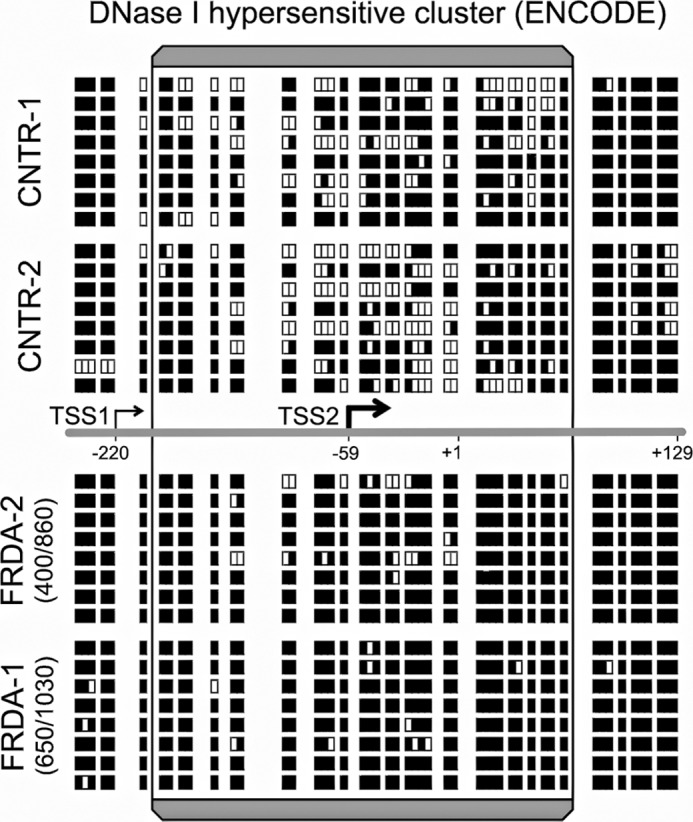
**Altered nucleosome positioning in the vicinity of the major *FXN*-TSS and obliteration of the naturally occurring NDR in FRDA.** NOMe-Seq data, measuring the nucleosomal footprint *in vivo*, for two non-FRDA (CNTR) and two FRDA lymphoblast cell lines are shown *above* and *below the line* depicting the region in the vicinity of *FXN*-TSS2 (the major *FXN*-TSS). The *boxed* region corresponds to the DNase I-hypersensitive cluster or NDR per ENCODE, within which TSS2 maps, consistent with its location in a region of relatively open chromatin configuration. NOMe-Seq data from eight clones each for the four cell lines analyzed is depicted in the form of *white* and *black boxes*, each of which represents accessible and inaccessible GpC sites, respectively (see “Experimental Procedures”). The region around TSS2 in the two non-FRDA cell lines shows many accessible GpC sites, indicating that it is in a NDR. In both FRDA cell lines, the region around TSS2 is largely inaccessible, indicating nucleosomal repositioning and obliteration of the naturally occurring NDR. Interestingly, TSS1, which maps just outside the NDR, is equally inaccessible in non-FRDA and FRDA cell lines.

To determine the mechanism of reduced chromatin accessibility at the *FXN*-TSS in FRDA, the region spanning the NDR was analyzed using NOMe-Seq, a high-resolution *in vivo* footprint assay for nucleosome occupancy (19). In the NOMe-Seq assay, inaccessible GpC dinucleotides indicate protection by nucleosomes (and vice versa), and sequencing of individual cloned PCR products thus reveals the pattern of nucleosome occupancy in individual chromatin molecules. The NOMe-Seq assay was especially suitable because of the high density of GpC dinucleotides in the vicinity of the two reported *FXN*-TSS (positions −59 and −220). Eight individual clones were thus analyzed from each of two FRDA and two non-FRDA cell lines (Fig. 3; *white* and *black boxes* indicate accessible and inaccessible GpC dinucleotides, respectively). As expected, *FXN*-TSS2 mapped within an NDR in non-FRDA control cell lines (Fig. 3; *upper panels*). In contrast, FRDA patients showed a dramatic loss of accessibility at *FXN*-TSS2, with almost no accessible GpC dinucleotides (Fig. 3; *lower panels*). This indicates that inaccessibility of *FXN*-TSS2 in FRDA is caused by altered nucleosome positioning at the NDR. *FXN*-TSS1, which is not active in lymphoblasts (11), was found to map just outside the NDR despite its close physical proximity to *FXN*-TSS2 and was equally inaccessible in non-FRDA and FRDA cell lines (Fig. 3). Taken together, our data indicate that *FXN*-TSS2, the major *FXN*-TSS, is much less accessible in FRDA due to altered nucleosome positioning and obliteration of the naturally occurring NDR.

Because the NOMe-Seq protocol uses bisulfite conversion, it is also possible to simultaneously detect naturally occurring CpG methylation. However, no endogenous CpG methylation was detected in either the two FRDA or two non-FRDA cell lines in the vicinity of the two *FXN*-TSS.

##### Reduced Transcriptional Permissiveness of the FXN Promoter in FRDA

**FIGURE 4.**
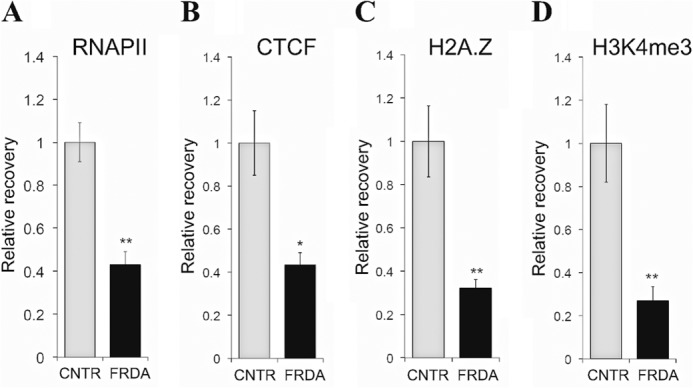
**Reduced transcriptional permissiveness of the *FXN* promoter in FRDA.** ChIP showing reduced occupancy of RNAPII (*A*) and CTCF (*B*), and depletion of H2A.Z (*C*) and H3K4me3 (*D*) in FRDA *versus* non-FRDA (CNTR) cells, indicating a state of reduced transcriptional permissiveness. RNAPII, H2A.Z and H3K4me3 ChIP was performed at E1 (Fig. 1*A*) and CTCF ChiP was performed in a region spanning *FXN*-TSS2 (between E1 and U1 in Fig. 1*A*) as described previously (17). These data represent the cumulative results from two complete experiments using three FRDA and three non-FRDA (CNTR) lymphoblast cell lines, with each assayed in triplicate. *Error bars* represent ± S.E. *, *p* < 0.05; **, *p* < 0.01.

The transcriptional permissiveness of typical human CpG island promoters is characterized by a well positioned +1 nucleosome preceded by a NDR (18) that is preassociated with RNAPII and the chromatin insulator CTCF (20, 21). Active genes are typically enriched for the transcriptionally permissive histone modification, H3K4me3, and the histone variant, H2A.Z, both of which are highly pronounced at the +1 nucleosome (21–23). We sought to determine whether the repressive chromatin and reduced accessibility of the *FXN*-TSS in FRDA had resulted in switching of the *FXN* promoter to a transcriptionally non-permissive state. Markers of transcriptional permissiveness at the *FXN*-TSS and the +1 nucleosome were assessed by chromatin immunoprecipitation in three FRDA and three non-FRDA cell lines. Indeed, the *FXN*-TSS in FRDA was found to be in a state of reduced transcriptional permissiveness as evidenced by reduced occupancy of RNAPII and CTCF (Fig. 4, *A* and *B*), and depletion of H2A.Z and H3K4me3 (Fig. 4, *C* and *D*). These data are consistent with observations previously made by us (17) and others (11, 24).

##### Deficient FXN Transcriptional Initiation in FRDA

**FIGURE 5.**
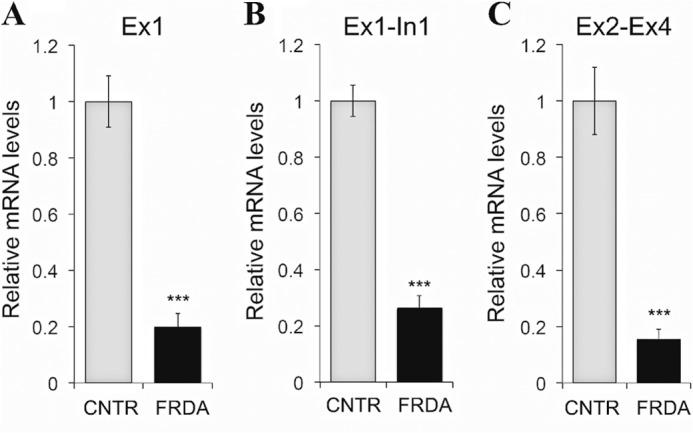
**Reduced transcriptional activity upstream of the expanded GAA-TR in FRDA.** *A* and *B*, quantitative RT-PCR showing significantly reduced amounts of *FXN* mRNA (Ex1 region (*A*) in Fig. 1*A*) and pre mRNA (Ex1-In1 region (*B*) in Fig. 1*A*) upstream of the GAA-TR in FRDA *versus* non-FRDA (CNTR) lymphoblast cells. *C*, quantitative RT-PCR showing a similar deficiency of *FXN* mRNA downstream of the GAA-TR in FRDA (Ex2-Ex4 region in Fig. 1*A*). Graphs represent the cumulative data from two complete experiments using three FRDA and three non-FRDA (CNTR) lymphoblast cell lines, each assayed in triplicate. *Error bars* represent ± S.E. ***, *p* < 0.001.

Given the repressive chromatin at *FXN*-TSS and its switch to a non-permissive transcriptional state in FRDA, we reasoned that this should lead to transcriptional deficiency even upstream of the expanded GAA-TR. To test this, quantitative RT-PCR was performed to measure steady-state levels of *FXN* transcript both upstream (exon 1 (Ex1 in Fig. 1*A*) and the exon 1-intron 1 junction (Ex1-In1 in Fig. 1*A*)) and downstream (spliced product of exons 2–4 (Ex2-Ex4 in Fig. 1*A*)) of the GAA-TR in three FRDA and three non-FRDA cell lines. FRDA patients showed a deficiency of steady-state levels of *FXN* transcript, both upstream (Fig. 5, *A* and *B*) and downstream (Fig. 5*C*) of the expanded GAA-TR. The reduction of transcriptional activity upstream of the expanded GAA-TR suggested that the *FXN* promoter is less active in FRDA and further suggested that transcriptional deficiency was not simply due to a defect in transcriptional elongation.

**FIGURE 6.**
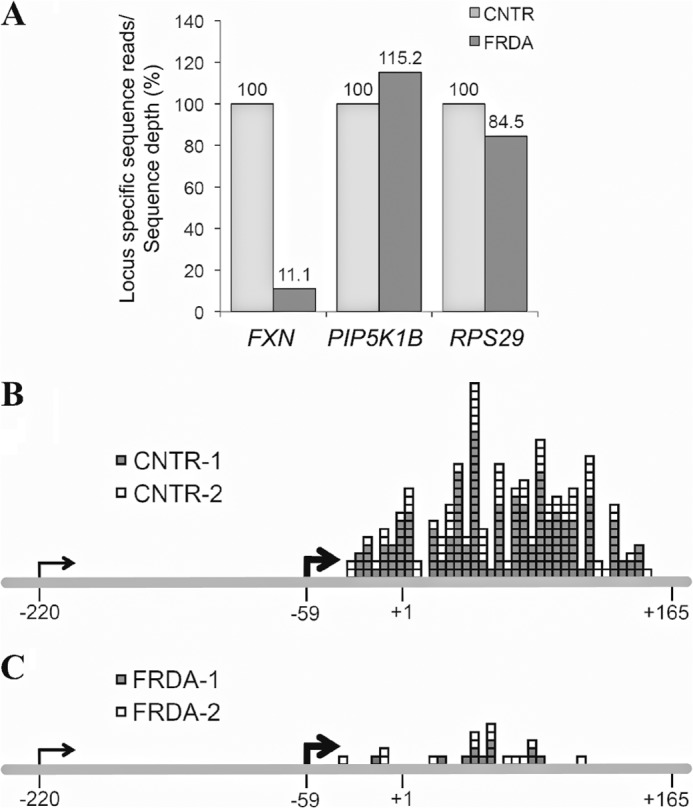
**Transcriptome (RNA-Seq) analysis showing deficiency of transcriptional initiation in FRDA.** *A*, RNA-Seq hits spanning the entire *FXN* gene showed 11.1% of normal in FRDA *versus* non-FRDA (CNTR) cell lines. No such difference was noted in the *PIP5K1B* gene, located immediately upstream of the *FXN* gene, or in *RPS29*, an unlinked housekeeping gene. Data are corrected for minor variations in sequence depth (average per cell line = 2 × 26 million reads) in each of two FRDA and two non-FRDA cell lines, with the non-FRDA cells depicted as 100%. *B* and *C*, RNA-Seq showing considerably less *FXN* mRNA sequence reads in the region upstream of the GAA-TR in two FRDA *versus* two non-FRDA (CNTR) lymphoblast cell lines (9.5% of normal; 25 *versus* 262). Each *box* indicates the 5′ end of a 100-bp sequence read (+165 denotes the 3′ end of exon 1). Note the lack of sequence reads between TSS1 (−220) and TSS2 (−59), indicating that TSS2 is the major *FXN*-TSS.

We next performed a complete transcriptome analysis (RNA-Seq) of two FRDA and two non-FRDA cell lines. Poly-A enrichment, and strand-specific library construction was followed by massively parallel, paired end sequencing using the Illumina HiSeq platform. Analysis of all hits spanning the *FXN* locus showed only 11.1% of normal (corrected for sequence depth; average per cell line = 2 × 26 million reads) in FRDA *versus* non-FRDA cell lines (Fig. 6*A*). No such difference was noted in the *PIP5K1B* gene, located immediately upstream of the *FXN* gene, or in *RPS29*, an unlinked housekeeping gene, indicating that transcriptional deficiency is limited to the *FXN* locus (Fig. 6*A*). Analysis of sequence reads specifically at the 5′ end of the *FXN* gene (*i.e.* including both *FXN*-TSS, the 5′-UTR, and exon 1) revealed several matches downstream of *FXN*-TSS2 (at −59) and none between *FXN*-TSS1 and *FXN*-TSS2 in the two control cell lines, confirming that *FXN*-TSS2 is the only active TSS in lymphoblasts (Fig. 6*B*; each box represents the 5′ end of a 100-bp sequence read). FRDA cell lines showed far fewer matches, amounting to 9.5% (25 *versus* 262) of normal (Fig. 6*C*), further substantiating the reduced amount of transcriptional activity upstream of the expanded GAA-TR, and indicating that the *FXN* promoter is much less active in FRDA.

**FIGURE 7.**
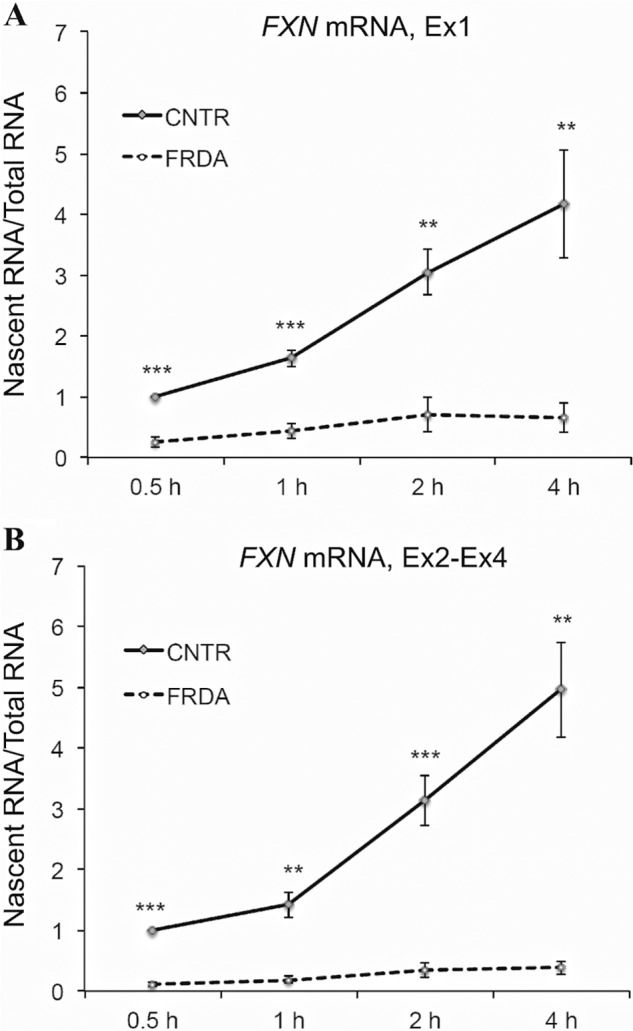
**Metabolic labeling of nascent *FXN* transcript in living cells showing deficiency of transcriptional initiation in FRDA.** *A* and *B*, quantitative RT-PCR of metabolically labeled nascent transcript for the indicated incubation times (0.5–4 h) is shown for *FXN* mRNA upstream (Ex1 region in Fig. 1*A*) and downstream (Ex2-Ex4 region in Fig. 1*A*) of the GAA-TR in intron 1. FRDA cells showed 5–12-fold less nascent *FXN* mRNA compared with non-FRDA cells (CNTR) at all time points assayed. Graphs represent the cumulative data from two complete experiments using two FRDA and two non-FRDA (CNTR) lymphoblast cell lines, each assayed in triplicate. *Error bars* represent ± S.E. **, *p* < 0.01; ***, *p* < 0.001.

To directly test whether the *FXN* promoter is rendered less active in FRDA, *FXN* transcriptional initiation was measured via metabolic labeling of nascent transcripts in two FRDA and two non-FRDA cell lines. Nascent transcripts were labeled with ethynyl uridine, to which biotin was subsequently added via click chemistry, thus permitting a quantitative, dynamic, *in vivo* analysis of the newly synthesized *FXN* transcript. This is similar to a nuclear run-on assay, with the added advantage of measuring nascent transcripts in live cells. Quantitative RT-PCR was performed to measure *FXN* transcript levels both upstream (Ex1, which maps immediately downstream of *FXN*-TSS2 (Fig. 1*A*)) and downstream (Ex2-Ex4 (Fig. 1*A*)) of the expanded GAA-TR following 0.5, 1, 2, and 4 h of labeling. This revealed a severe (5–12-fold) deficiency of newly synthesized *FXN* transcript in FRDA at both locations (Fig. 7, *A* and *B*). The deficiency of dynamic accumulation of *FXN* transcript levels noted upstream of the expanded GAA-TR (but immediately downstream of *FXN*-TSS2) indicated a severe deficiency of transcriptional initiation in FRDA (note that the half-life of *FXN* transcript remains unchanged in FRDA (10)). Comparison of the levels of newly synthesized *FXN* transcript upstream *versus* downstream of the expanded GAA-TR revealed a consistent ∼2-fold decrease in the downstream location, indicating that transcriptional elongation further contributes to the transcriptional deficiency in FRDA. However, the 5–12-fold reduction in newly synthesized *FXN* mRNA upstream of the expanded GAA-TR indicates that deficient transcriptional initiation, and not elongation, is the predominant cause of *FXN* transcriptional deficiency in FRDA.

## DISCUSSION

**FIGURE 8.**
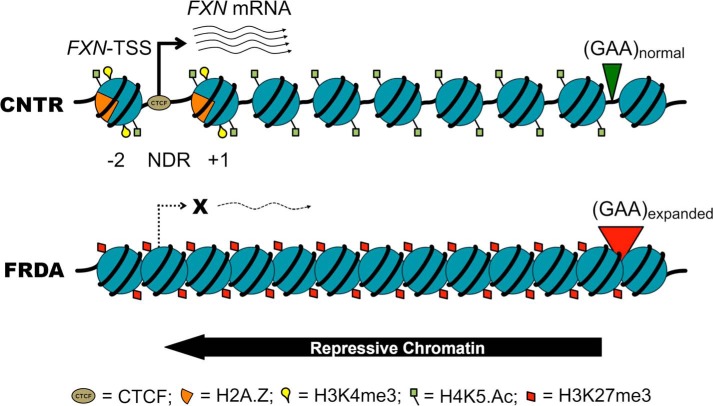
**Model of deficient transcriptional initiation in FRDA.** The expanded GAA-TR mutation in FRDA serves as a source of repressive chromatin (indicated here as trimethylation of H3K27 and hypoacetylation of H4K5), which spreads to the upstream regions of the *FXN* gene to involve the major *FXN*-TSS located 1.6 kb upstream. The major *FXN*-TSS, which is normally in a NDR, is rendered inaccessible because of increased nucleosome density. This results in a *FXN* promoter that is transcriptionally non-permissive (indicated here as obliteration of the NDR and depletion of CTCF, H3K4me3, and H2A.Z), leading to a severe deficiency of transcriptional initiation in FRDA.

We show that the expanded GAA-TR mutation in FRDA, located 1.6 kb away from the *FXN*-TSS results in disruption of its transcription-permissive chromatin structure and causes a deficiency of transcriptional initiation. Our data are consistent with the model that in FRDA, there is spread of repressive chromatin from the expanded GAA-TR in intron 1 to the upstream region of the *FXN* gene (Fig. 8). This spread results in alteration of nucleosome positioning, reduced accessibility of the *FXN*-TSS, which is normally in an open chromatin configuration, and severely deficient *FXN* transcriptional initiation (Fig. 8). As a consequence of reduced transcriptional initiation in FRDA, there is significantly diminished *FXN* transcriptional activity, detectable both upstream and downstream of the expanded GAA-TR. Notably, these findings are congruent with those of Saveliev *et al.* (5), who found that an expanded triplet repeat sequence (GAA or CTG) resulted in variegated silencing of a linked transgenic reporter gene. Indeed, silencing was mediated by an increase in nucleosomal density leading to a closed chromatin configuration of a nearby heterologous promoter, which is similar to what we have observed at the *FXN* promoter in FRDA.

A widely accepted model of the mechanism of transcriptional deficiency in FRDA is that there is deficient transcriptional elongation through the expanded GAA-TR in intron 1. This is predicated on the presence of repeat-proximal heterochromatin (6–9) and its reversal via a histone deacetylase inhibitor that reverses *FXN* transcriptional deficiency in FRDA (6, 25) and the ability of the expanded repeat to adopt secondary structures that can interfere with transcriptional elongation (3, 14–16, 26, 27). Indeed, some have even questioned the existence of any other transcriptional defect, claiming that the transcriptional defect in FRDA is due solely to deficient transcriptional elongation (10). However, our present data, along with those of Kumari *et al.* (11), confirm the existence of deficient transcriptional initiation in FRDA. In fact, the transcriptional initiation defect, which is related to the spread of repressive chromatin from the expanded GAA-TR mutation, seems to be the predominant contributor to the overall deficiency of *FXN* transcript in FRDA.

Several questions remain unanswered. For instance, what is the typical distance over which an expanded triplet repeat exerts its transcriptional silencing effect? Is the extent of spread or strength of repression related to repeat length? Answers to these questions would help uncover the mechanism of the known length-dependence of transcriptional deficiency (9, 13, 28) and severity of clinical phenotype (29, 30) in FRDA. There are numerous polymorphic GAA triplet repeat tracts in the genomes of mammals, many of which are located within genes, and some are of considerable length (31). It is plausible that these polymorphic repeats could serve as localized and allele-specific epigenetic regulators of transcription. Furthermore, it remains unclear if the repressive chromatin at the *FXN*-TSS in FRDA is variegated. Our NOMe-Seq data showed obliteration of the NDR in almost every chromatin fiber analyzed, which would tend to support the absence of variegation. However, a definitive answer would require sequencing of far more chromatin fibers than we have performed here, including chromatin associated with shorter than the typical expanded GAA alleles in FRDA. Another interesting unanswered question is what stops the spread of repressive chromatin emanating from an expanded triplet repeat? CTCF bound sequences are known to serve as chromatin insulators, some of which function as barriers to the spread of heterochromatin (32). However, CTCF does not seem to serve such a protective function in FRDA in the presence of the expanded GAA-TR. Not only is CTCF depleted from its binding site, which overlaps *FXN*-TSS2, but repressive chromatin in FRDA is established at *FXN*-TSS2 and also seems to spread further upstream. Among its many functions, CTCF is known to position nucleosomes (33), so it is also possible that CTCF depletion may have further contributed to the altered nucleosomal positioning in FRDA. An important caveat of our study is that it is based on findings from patient-derived lymphoblasts, which is not a cell type that is clinically affected in FRDA; clearly, additional confirmatory studies need to be carried out using more disease-appropriate cell types and/or tissues.

Finally, given the relative importance of deficient transcriptional initiation in FRDA, we propose that drugs that are designed to reactivate *FXN* transcriptional initiation would serve as a particularly efficient therapeutic strategy for FRDA.

## Footnotes

2 The abbreviations used are:

FRDA: Friedreich ataxia
TSS: transcriptional start site(s)
NDR: nucleosome-depleted region
TR: triplet repeat
CTCF: CCCTC binding factor
RNAP: RNA polymerase.
